# Association of Prognostic Understanding With Health Care Use Among Older Adults With Advanced Cancer: A Secondary Analysis of a Cluster Randomized Clinical Trial

**DOI:** 10.1001/jamanetworkopen.2022.0018

**Published:** 2022-02-18

**Authors:** Kah Poh Loh, Christopher L. Seplaki, Chandrika Sanapala, Reza Yousefi-Nooraie, Jennifer L. Lund, Ronald M. Epstein, Paul R. Duberstein, Marie Flannery, Eva Culakova, Huiwen Xu, Colin McHugh, Heidi D Klepin, Po-Ju Lin, Erin Watson, Valerie Aarne Grossman, Jane Jijun Liu, Jodi Geer, Mark A. O’Rourke, Karen Mustian, Supriya G. Mohile

**Affiliations:** 1 James P Wilmot Cancer Institute, Division of Hematology/Oncology, Department of Medicine, University of Rochester Medical Center, Rochester, New York; 2 Department of Public Health Sciences, University of Rochester School of Medicine and Dentistry, Rochester, New York; 3 Department of Epidemiology, Gillings School of Global Public Health, University of North Carolina at Chapel Hill; 4 Lineberger Comprehensive Cancer Center, University of North Carolina at Chapel Hill; 5 Center for Communication and Disparities Research, Department of Family Medicine, University of Rochester School of Medicine and Dentistry, Rochester, New York; 6 Department of Medicine, Palliative Care, University of Rochester School of Medicine and Dentistry, Rochester, New York; 7 Department of Health Behavior, Society, and Policy, Rutgers School of Public Health, Piscataway, New Jersey; 8 School of Nursing, University of Rochester School of Medicine and Dentistry, Rochester, New York; 9 Department of Surgery, Cancer Control, University of Rochester School of Medicine and Dentistry, Rochester, New York; 10 Department of Preventive Medicine and Population Health, School of Medicine, Sealy Center on Aging, University of Texas Medical Branch, Galveston; 11 Section on Hematology and Oncology, Wake Forest Baptist Comprehensive Cancer Center, Winston-Salem, North Carolina; 12 Princeton University, Princeton, New Jersey; 13 SCOREboard Advisory Group, University of Rochester Medical Center, Rochester, New York; 14 Heartland National Cancer Institute Community Oncology Research Program (NCORP), Decatur, Illinois; 15 Metro Minnesota Community Oncology Research Program, St Louis Park; 16 NCORP of the Carolinas (Greenville Health System NCORP), Greenville, South Carolina; 17 James P. Wilmot Cancer Center, Department of Medicine, University of Rochester, Rochester, New York

## Abstract

**Importance:**

A poor prognostic understanding regarding curability is associated with lower odds of hospice use among patients with cancer. However, the association between poor prognostic understanding or prognostic discordance and health care use among older adults with advanced incurable cancers is not well characterized.

**Objective:**

To evaluate the association of poor prognostic understanding and patient-oncologist prognostic discordance with hospitalization and hospice use among older adults with advanced cancers.

**Design, Setting, and Participants:**

This was a post hoc secondary analysis of a cluster randomized clinical trial that recruited patients from October 29, 2014, to April 28, 2017. Data were collected from community oncology practices affiliated with the University of Rochester Cancer Center National Cancer Institute Community Oncology Research Program. The parent trial enrolled 541 patients who were aged 70 years or older and were receiving or considering any line of cancer treatment for incurable solid tumors or lymphomas; the patients’ oncologists and caregivers (if available) were also enrolled. Patients were followed up for at least 1 year. Data were analyzed from January 3 to 16, 2021.

**Main Outcomes and Measures:**

At enrollment, patients and oncologists were asked about their beliefs regarding cancer curability (100%, >50%, 50%, <50%, and 0%; answers other than 0% reflected poor prognostic understanding) and life expectancy (≤6 months, 7-12 months, 1-2 years, 2-5 years, and >5 years; answers of >5 years reflected poor prognostic understanding). Any difference between oncologist and patient in response options was considered discordant. Outcomes were any hospitalization and hospice use at 6 months captured by the clinical research associates.

**Results:**

Among the 541 patients, the mean (SD) age was 76.6 (5.2) years, 264 of 540 (49%) were female, and 486 of 540 (90%) were White. Poor prognostic understanding regarding curability was reported for 59% (206 of 348) of patients, and poor prognostic understanding regarding life expectancy estimates was reported for 41% (205 of 496) of patients. Approximately 60% (202 of 336) of patient-oncologist dyads were discordant regarding curability, and 72% (356 of 492) of patient-oncologist dyads were discordant regarding life expectancy estimates. Poor prognostic understanding regarding life expectancy estimates was associated with lower odds of hospice use (adjusted odds ratio, 0.30; 95% CI, 0.16-0.59). Discordance regarding life expectancy estimates was associated with greater odds of hospitalization (adjusted odds ratio, 1.64; 95% CI, 1.01-2.66).

**Conclusions and Relevance:**

This study highlights different constructs of prognostic understanding and the need to better understand the association between prognostic understanding and health care use among older adult patients with advanced cancer.

**Trial Registration:**

ClinicalTrials.gov Identifier: NCT02107443

## Key Points

**Question:**

What is the association of poor prognostic understanding and patient-oncologist prognostic discordance with health care use among older adult patients with advanced incurable cancer?

**Findings:**

In this post hoc secondary analysis of a cluster randomized clinical trial with 541 participants, a poor prognostic understanding regarding life expectancy estimates was associated with lower odds of hospice use. Patient-oncologist discordance regarding life expectancy estimates was associated with greater odds of hospitalization.

**Meaning:**

This study highlighted different constructs of prognostic understanding and the need to better understand the associations between prognostic understanding and health care use among older adult patients with advanced incurable cancer.

## Teaser

This post hoc secondary analysis of a cluster randomized clinical trial evaluates the association of poor prognostic understanding and patient-oncologist prognostic discordance with hospitalization and hospice use among older adults with advanced cancers.

## Introduction

*Prognostic understanding* refers to the perception of prognosis (ie, curability or life expectancy estimates). Patients with incurable cancers who perceive their cancers as curable or overestimate their survival may be more willing to accept life-extending therapy,^1,2^ may have a lower likelihood of having a do-not-resuscitate order,^3^ and may be less likely to receive or want hospice care at the end of life.^4,5^ Therefore, ensuring that patients with advanced cancers have an accurate understanding of their prognosis (or promoting acceptance of their prognosis) is essential for treatment decision-making, advance care planning, and psychological support.

Various methods have been used to assess prognostic understanding. Many studies have assessed the prognostic understanding only of patients,^6^ although some have also included oncologist and caregiver assessments.^7,8^ Those that focused only on patient understanding often used the term *poor prognostic understanding* or *poor prognostic awareness*, while those evaluating prognostic understanding between 2 people (eg, patients vs oncologists, patients vs caregivers, or oncologists vs caregivers) used the term *prognostic discordance* or *prognostic disagreement*, indicating differences between 2 individuals. Prognostic understanding, whether of the patient only or in the evaluation of prognostic discordance between 2 people, is commonly assessed regarding curability (or treatment goals that include curability) and life expectancy estimates.

More than 60% of patients with a new diagnosis of cancer are aged 65 years or older, and approximately 70% of all cancer deaths occurred among adults aged 65 years or older.^9^ Many older adults have age-related conditions, such as functional and cognitive impairments, that may influence communication, thereby affecting prognostic understanding and prognostic discordance. Studies have also shown that older adults have a lower awareness of their diagnosis and prognosis than their younger counterparts.^10,11,12^ There are also oncologist-related factors associated with prognostic communication and understanding (eg, lack of confidence in responding to patients’ emotions during prognostic discussion and overestimation of prognosis).^8,13^ Despite these findings, to our knowledge, few studies have focused on prognostic understanding among older adults and prognostic discordance between older adults and clinicians.^8,14,15,16^ Furthermore, the association between poor prognostic understanding or prognostic discordance and health care use among older adults with advanced incurable cancers is not well characterized.^15^ Studies have shown that disagreements between patient and physician in various aspects of care can be associated with negative outcomes, such as diminished trust in the physician, dissatisfaction with the visit, lack of adherence to treatment, higher rates of hospitalization, and increased risk of death.^17,18,19,20^

In this study, we focused on prognostic understanding in older adults with advanced cancer. Specifically, we aimed to evaluate (1) the prevalence of poor prognostic understanding and prognostic discordance, (2) the association between prognostic understanding and prognostic discordance, and (3) the association of poor prognostic understanding and prognostic discordance with hospitalization and hospice use.

## Methods

### Study Design, Setting, and Participants

We performed a post hoc secondary analysis of a cluster randomized clinical trial that assessed the effect of geriatric assessment (GA) and GA-guided recommendations on communication and satisfaction among older adult patients with advanced cancer, their caregivers, and oncologists (ClinicalTrials.gov: NCT02107443).^21^ Results from the parent study have been published previously, including the study flow diagram, methods, types of randomization, assessments of the primary and secondary outcomes, and determination of sample size.^21^ The primary study was conducted within the University of Rochester Cancer Center National Cancer Institute Community Oncology Research Program Research Base Network. The primary study recruited patients who were aged 70 years or older, had a diagnosis of an incurable stage III or IV solid tumor or lymphoma, had at least 1 impaired GA domain, and were considering or receiving any kind of cancer treatment. The study did not specifically define *incurable*, and the definition was based on the oncologist’s judgment. The patient’s oncologist and caregiver (if available) were also enrolled in the study. A total of 31 community oncology practices participated in the study between October 29, 2014, and April 28, 2017 (eTable 1 in Supplement 1). The institutional review boards of the University of Rochester and all participating sites approved this study. All participants provided written informed consent. This report follows the Consolidated Standards of Reporting Trials (CONSORT) reporting guideline for randomized clinical trials.^22^

### Primary Exposures: Poor Prognostic Understanding and Prognostic Discordance

**Figure 1.**
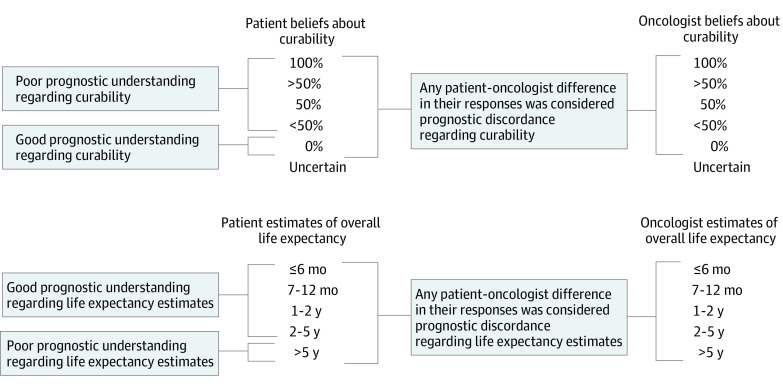
Definitions of Poor Prognostic Understanding and Prognostic Discordance Definitions of poor prognostic understanding and prognostic discordance regarding curability and life expectancy estimates, as well as prognostic discordance regarding curability and life expectancy estimates, are shown. For poor patient prognostic understanding regarding curability, note that patients with a response of uncertain were excluded. For prognostic discordance regarding curability, patients and oncologists with a response of uncertain were excluded.

At enrollment, patients and oncologists were asked about their beliefs about the curability of the cancer: “What do you believe are the chances the cancer will go away and never come back with treatment?” Response options were 100%, more than 50%, 50%, less than 50%, 0%, or uncertain. Patients and oncologists were also asked: “Considering your (the patient’s) health, and your (the patient’s) underlying medical conditions, what would you estimate your (the patient’s) overall life expectancy to be?” Response options were 6 months or less, 7 to 12 months, 1 to 2 years, 2 to 5 years, and more than 5 years. Both questions were adapted from prior studies.^7,23^ Any response other than 0% for curability was considered poor prognostic understanding regarding curability (answers of uncertain were excluded). Response of life expectancy more than 5 years was considered poor prognostic understanding regarding life expectancy estimates (Figure 1). We chose this most conservative definition because the study population of older adults had a variety of advanced cancers (median survival from 6 months to >5 years depending on the cancer type).^24,25,26^

Prognostic discordance regarding curability was defined as the presence of any patient-oncologist difference in their numerical responses; missing or uncertain responses were excluded from this analysis. Similar definitions were used to establish prognostic discordance regarding life expectancy estimates (uncertain was not a response option) (Figure 1).

### Outcome Variables: Any Hospitalization and Hospice Use

Any hospitalization for any reason within the first 6 months of enrollment was captured by the clinical research associates at each site. Clinical research associates completed forms at 4 to 6 weeks, 3 months, and 6 months. When available, dates of hospitalization were captured. We also collected data on hospice use and dates of hospice, if available, for the first 6 months. Although patients could withdraw from the study at any time, data on hospitalization and hospice use were collected unless patients signed a written form informing the study team that they no longer wanted their health care data to be collected.

### Covariates

Covariates included patient demographic information (age, sex, race and ethnicity, educational level, marital status, and annual household income), clinical data (cancer types), and age-related conditions as measured using a GA. Race and ethnicity was self-reported by participants, and options were predefined by the investigator. Data on race and ethnicity were collected to better understand the characteristics of the participants and whether they were associated with outcomes of interest. The GA domains included comorbidity, functional status, physical performance, cognition, instrumental social support, polypharmacy, psychological health, and nutrition. These GA domains have been described previously.^27^ The GA domains were assessed using validated tools and thresholds for impairment. Patients completed the self-reported portions of the GA. Clinical research associates performed the objective assessment portions of the GA. We included these covariates because they may be associated with prognostic understanding as well as with health care use.^14,28,29,30,31,32,33,34,35,36,37^

### Statistical Analysis

Statistical analysis was conducted from January 3 to 16, 2021. After describing the population and prognostic understanding and prognostic discordance using descriptive analyses, we used the χ^2^ test to evaluate the association between prognostic understanding and prognostic discordance. We used the *t* test (for continuous variables) and the χ^2^ test or the Fisher exact test (for categorical variables) to assess whether poor prognostic understanding or prognostic discordance and the individual covariates were associated with hospitalization and use of hospice. We then used generalized estimating equations with a logistic model specification to estimate the bivariate and multivariable associations; the latter were adjusted for demographic characteristics, cancer type, study group, and age-related conditions and accounting for clustering at the practice level. We created 4 separate models for each outcome (ie, poor prognostic understanding regarding curability and life expectancy estimates and prognostic discordance regarding curability and life expectancy estimates). Uncertain responses were not included in the curability models because we examined discordance using numerical data.

We performed several sensitivity analyses. First, for prognostic understanding regarding curability, we examined the odds ratio (OR) and 95% CI comparing individual response options with a response of 0% (responses of uncertain were excluded). For prognostic understanding regarding life expectancy estimates, we examined the OR and 95% CI comparing individual response options with a response of 6 months or less. These analyses allowed us to examine dose-response associations for both hospitalization and hospice use. Second, for prognostic discordance, we collapsed our exposure variables in the following ways: (1) beliefs about curability (0% vs >0%-100%) and (2) life expectancy estimates (≤12 months vs 1-2 years vs 2 to >5 years). We then redefined prognostic discordance regarding curability and life expectancy estimates if there was any patient-oncologist difference in the response options, and we repeated the generalized estimating equation models. All *P* values were from 2-sided tests, and the results were considered statistically significant at *P* < .05. All statistical analyses were conducted using the SAS software package, version 9.3 (SAS Institute Inc).

## Results

### Demographics and Clinical Variables

The primary cluster randomized trial included 541 patients. The characteristics of the sample have been described previously.^27^ In brief, the mean (SD) patient age was 76.6 (5.2) years, 264 of 540 (49%) were female, 486 of 540 (90%) were White, and 279 of 540 (52%) had at least some college education (eTable 2 in Supplement 1). The most common cancer types were lung (137 of 540 [25%]) and gastrointestinal cancer (129 of 540 [24%]).

### Prognostic Understanding Regarding Curability and Life Expectancy Estimates

**Figure 2.**
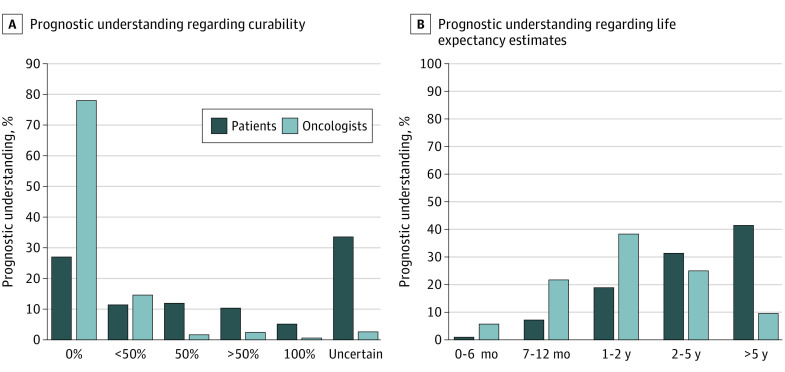
Distribution of Prognostic Understanding Regarding Curability and Life Expectancy Estimates Reported by Patients and Oncologists A, Patients and oncologists were asked about their beliefs about the curability of the cancer: “What do you believe are the chances the cancer will go away and never come back with treatment?” Any response other than 0% for curability was considered poor prognostic understanding regarding curability (59% [206 of 348]; responses of uncertain were excluded). B, Patients and oncologists were asked: “Considering your (the patient’s) health, and your (the patient’s) underlying medical conditions, what would you estimate your (the patient’s) overall life expectancy to be?” Response of life expectancy of more than 5 years was considered poor prognostic understanding regarding life expectancy estimates (41% [205 of 496]).

Figure 2A and B show the distribution of prognostic understanding regarding curability and life expectancy estimates by both patients and oncologists. Prognostic understanding regarding curability by patients has been reported previously (Figure 2A).^14^ Of the 348 patients who provided numerical responses for curability, 206 (59%) demonstrated poor prognostic understanding regarding curability.^14^ Poor prognostic understanding regarding life expectancy estimates was reported for 41% of patients (205 of 496). Table 1 shows the agreement between patients and oncologists within the individual response options.

**Table 1.** Patient and Oncologist Beliefs About Curability and Estimates of Overall Life Expectancy

Oncologist beliefs about curability, % (N = 521)^a^						
Patient beliefs about curability	100%	>50%	50%	<50%	0%	Uncertain
100%	0	0	0.4	1.3	3.3	0.4
>50%	0.2	1.5	0.8	2.1	5.0	0.6
50%	0	0.4	0	2.7	8.8	0.2
<50%	0	0.2	0	1.3	10.0	0.2
0%	0	0.2	0.2	3.3	22.8	0.6
Uncertain	0	0.4	0.4	4.0	27.8	1.0
**Oncologist estimates of overall life expectancy, % (N = 492)^a^**						
Patient estimates of overall life expectancy	0-6 mo	7-12 mo	1-2 y	2-5 y	>5 y	NA
≤6 mo	0.2	0.8	0.2	0	0	NA
7-12 mo	1.0	2.9	2.4	0.6	0.4	NA
1-2 y	0.8	6.9	8.5	1.8	0.6	NA
2-5 y	0.8	4.7	13.2	9.8	2.9	NA
>5 y	1.8	5.9	14.0	13.4	6.3	NA

Abbreviation: NA, not applicable.

^a^ Missing responses from either oncologist or patient were excluded.

Among dyads who provided numerical responses, prognostic discordance regarding curability occurred in 60% (202 of 336), and prognostic discordance regarding life expectancy estimates occurred in 72% (356 of 492). Most discordances were due to patients estimating a higher chance of curability (89% [179 of 202]) and greater life expectancy (87% [308 of 356]) than oncologists.

### Association Between Prognostic Understanding Regarding Curability and Life Expectancy Estimates

There was an association between prognostic understanding regarding curability and life expectancy. Among the 206 patients with poor prognostic understanding regarding curability, 106 of 199 (53%; 7 patients had missing data) also had poor prognostic understanding regarding life expectancy estimates (*P* < .001, assessed by use of the χ^2^ test). Among the 205 patients with poor prognostic understanding regarding life expectancy estimates, 106 of 133 (80%; 72 patients had missing data mostly due to selecting uncertain response) also had poor prognostic understanding regarding curability (*P* < .001, assessed by use of the χ^2^ test).

### Association Between Prognostic Understanding and Prognostic Discordance

For curability, poor prognostic understanding was positively associated with prognostic discordance (Table 2). Among the 19 patients who believed their curability to be 0% and were discordant with their oncologist, 17 oncologists believed the patient’s cancer curability to be less than 50%, 1 believed it to be 50%, and 1 believed it to be more than 50% (*P* < .001, assessed by use of the χ^2^ test).

**Table 2.** Associations Between Prognostic Understanding and Prognostic Discordance Regarding Curability and Life Expectancy Estimates

Estimate	Discordance	Concordance	*P* value^a^
Patient estimates of curability			
No.	202	134	NA
0%	19 (9)	119 (89)	<.001
<50%	53 (26)	7 (5)	
50%	62 (31)	0	
>50%	42 (21)	8 (6)	
100%	26 (13)	0	
Poor prognostic understanding regarding curability	183 (91)	15 (11)	
Do not have poor prognostic understanding regarding curability	19 (9)	119 (89)	
Life expectancy estimates by patients			
No.	356	136	NA
≥6 mo	5 (1)	1 (1)	<.001
7-12 mo	22 (6)	14 (10)	
1-2 y	50 (14)	42 (31)	
2-5 y	106 (30)	48 (35)	
>5 y	173 (49)	31 (23)	
Poor prognostic understanding regarding life expectancy estimates	173 (49)	31 (23)	
Do not have poor prognostic understanding regarding life expectancy	183 (51)	105 (77)	

Abbreviation: NA, not applicable.

^a^ Assessed by use of the χ^2^ test.

Similarly, for life expectancy estimates, poor prognostic understanding was positively associated with prognostic discordance. Among the 5 patients who estimated their life expectancy to be 6 months or less and were discordant with their oncologist, 4 oncologists estimated the patient’s life expectancy to be 7 to 12 months and 1 oncologist estimated it to be 1 to 2 years.

### Hospitalization and Hospice Use

Of the 541 patients, 130 (24%) were hospitalized within the first 6 months of enrollment (eTable 2 in Supplement 1). A total of 82 patients (15%) used hospice within the first 6 months of enrollment (eTable 3 in Supplement 1).

### Poor Prognostic Understanding, Prognostic Discordance, and Hospitalization

On bivariate analyses, poor prognostic understanding regarding life expectancy estimates was associated with lower odds of hospitalization (OR, 0.66; 95% CI, 0.48-0.89) (Table 3). On multivariable analyses, the association of poor prognostic understanding regarding life expectancy estimates and hospitalization was no longer significant (adjusted OR [AOR], 0.74; 95% CI, 0.50-1.08). Prognostic discordance regarding life expectancy estimates was associated with greater odds of hospitalization (AOR, 1.64; 95% CI, 1.01-2.66).

**Table 3.** Bivariate and Multivariable Associations of Poor Prognostic Understanding and Prognostic Discordance Regarding Curability and Survival Estimates With Hospitalization and Hospice Use

Outcome	Odds ratio (95% CI)	
	Bivariate analyses^a^	Multivariable analyses, adjusted^b^
Hospitalization		
Poor prognostic understanding regarding curability	0.71 (0.46-1.11)	0.77 (0.49-1.21)
Poor prognostic understanding regarding life expectancy estimates	0.66 (0.48-0.89)	0.74 (0.50-1.08)
Prognostic discordance regarding curability	0.80 (0.48-1.34)	0.87 (0.50-1.53)
Prognostic discordance regarding life expectancy estimates	1.50 (0.94-2.37)	1.64 (1.01-2.66)
Hospice use		
Poor prognostic understanding regarding curability	0.67 (0.48-0.92)	0.76 (0.51-1.12)
Poor prognostic understanding regarding life expectancy estimates	0.25 (0.13-0.47)	0.30 (0.16-0.59)
Prognostic discordance regarding curability	0.76 (0.54-1.07)	0.78 (0.50-1.22)
Prognostic discordance regarding life expectancy estimates	1.25 (0.84-1.86)	1.27 (0.73-2.20)

^a^ Accounting for clustering at the practice level.

^b^ Adjusted for demographic characteristics, cancer type, study group, and age-related conditions and accounting for clustering at the practice level.

### Poor Prognostic Understanding, Prognostic Discordance, and Hospice Use

On bivariate analyses, poor prognostic understanding regarding curability and life expectancy estimates were both associated with lower odds of hospice use (curability: OR, 0.67; 95% CI, 0.48-0.92; life expectancy: OR, 0.25; 95% CI, 0.13-0.47) (Table 3). On multivariable analyses, poor prognostic understanding regarding life expectancy estimates was associated with lower odds of hospice use (AOR, 0.30; 95% CI, 0.16-0.59).

In sensitivity analyses, a dose-response association was noted for the associations between life expectancy estimates and hospice use (eTable 4 in Supplement 1). Prognostic discordance regarding life expectancy estimates (after variables have collapsed and prognostic discordance was redefined) was associated with greater odds of hospitalization (eTable 5 in Supplement 1).

## Discussion

In this secondary analysis of more than 500 older adults with advanced incurable cancers, we found that poor prognostic understanding and prognostic discordance regarding curability and life expectancy estimates were associated but were not the same. Prognostic discordance regarding life expectancy estimates was associated with greater odds of hospitalization, while poor prognostic understanding regarding life expectancy estimates was associated with lower odds of hospice use; in multivariable models, these patterns were not observed regarding curability estimates.

Studies have used differing methods and definitions of prognostic understanding and prognostic discordance as well as different patient populations, yielding prevalences ranging from 0% to 96%.^6,38,39^ Therefore, comparison across studies is challenging. We have shown that between 41% and 72% of older adults had poor prognostic understanding. Other studies focusing on older adults have demonstrated a similar prevalence. In a single-center study^15^ of patients aged 70 years or older with advanced gastrointestinal cancers, poor prognostic understanding was assessed in the form of illness perceptions and treatment goals (or curability); 50.5% of patients reported a non–terminally ill status, and 36.0% reported a curative treatment goal. In a study of patients aged 60 years or older with acute myeloid leukemia at 2 institutions, prognostic discordance in the form of curability occurred in 67% of patient-oncologist dyads.^16^ Overall, studies suggest that poor prognostic understanding and prognostic discordance are common and context specific, and they are likely due to both the patient’s understanding of their illness (eg, not wanting to know their prognosis, fixed beliefs, or inaccurate interpretation of prognostic information) and the quality of communication between oncologist and patient (eg, avoidance of a prognostic discussion).^40,41^ In this sample of older patients with incurable advanced cancer, more than 20% of oncologists thought that there was at least some chance of curability, with 4.5% selecting 50% or more. Prior studies have shown that oncologists may have difficulty estimating prognosis, and their estimates were often more optimistic than actual patient survival, although their estimates tended to be more accurate than patients’ estimates.^42,43^

Studies on the association between prognostic understanding and hospitalization are lacking. Among older patients with advanced gastrointestinal cancers, those who had poor prognostic understanding (ie, those who selected non–terminally ill status) had a lower risk of hospitalization.^15^ Although not statistically significant (because the 95% CI crosses 1), our results also suggested that patients with poor understanding regarding life expectancy estimates may be less likely to be hospitalized. These patients perceived themselves as likely to live longer, and their positive perceptions may have been because of a better underlying health status, which might confer a lower risk of hospitalization. For more than 4500 older adults, a positive self-perception of aging (which likely is associated with survival expectations) in and of itself was also associated with a lower rate of hospitalization.^44^ We found that prognostic discordance regarding life expectancy estimates was associated with increased hospitalization, possibly reflecting poor communication associated with worse health status and patient preferences for more intensive treatment at the end of life.^45^

Poor prognostic understanding regarding curability has previously been shown to be associated with lower use of hospice.^1^ Similarly, we showed that poor prognostic understanding regarding life expectancy estimates was associated with lower odds of hospice use, which was further supported by a dose-response association. Although the association between prognostic understanding regarding curability and hospice use was not statistically significant on multivariable analyses, the directionality was similar. The lack of statistical significance may be because of insufficient sample size and follow-up. Some of our patients may have used hospice beyond the study follow-up period, which was not captured.

### Strengths and Limitations

This study had some strengths. The major strength was its inclusion of more than 500 older adults with advanced incurable cancer recruited from community oncology practices in the United States. Our study demonstrated differences between prognostic understanding and prognostic discordance regarding curability and life expectancy estimates. Although poor prognostic understanding and prognostic discordance regarding life expectancy estimates were positively associated, their associations with health care use differed, suggesting that prognostic understanding obtained from patients alone may be different from comparison of prognostic understanding between patients and oncologists. Given the high prevalence of poor prognostic understanding discordance, our findings also highlight the need to design multimodal interventions to address this discordance in older adults with advanced cancer. These interventions should aim at improving communication about prognosis with appropriate support provided.^23,46,47,48^

This study also has several limitations. First, we recruited primarily non-Hispanic White and well-educated patients. Based on prior studies, poor prognostic understanding and discordance were more prevalent in minority racial and ethnic populations.^7,8^ Second, given that we recruited a heterogeneous sample of older patients with advanced cancer, it was challenging to define poor prognostic understanding in the form of life expectancy estimates. Although we used a uniform method regardless of cancer type and the most conservative approach (ie, >5 years, as most older patients would not have lived beyond 5 years), it is likely that a larger number of patients could have been considered to have poor prognostic understanding (eg, those with pancreatic cancer who chose 2-5 years). Incurable cancer was one of the eligibility criteria for the study, but approximately 22% of oncologists selected a response other than 0% for curability. Reasons for this selection are unclear, but it is possible that oncologists want to retain a sense of hope and that there were insufficient response options (eg, 1%-10%) to capture this hopefulness, or that, in some cancers previously designated as incurable (eg, oligometastatic colon cancer), a small chance of curability may be possible. Third, while 1 oncologist-patient encounter was recorded for each patient, these encounters may not have focused on discussions of prognosis between patients and oncologists. Future work that investigates prognostic understanding should capture these important conversations. Fourth, we did not adjust for multiple comparisons given the exploratory nature of our study.

## Conclusions

Poor prognostic understanding in the form of life expectancy estimates was associated with lower odds of hospice use. Prognostic discordance in the form of life expectancy estimates was associated with greater odds of hospitalization. Our study highlighted different constructs of prognostic understanding and demonstrated the need to assess all of these components in future studies to better understand the associations between prognostic understanding and health care use.

## Notes

Supplement 1. **eTable 1.** National Cancer Institute Community Oncology Research Program (NCORP) Community Affiliate Partners **eTable 2. **Associations of Poor Prognostic Understanding, Prognostic Discordance, and Individual Covariates With Hospitalization at 6 Months **eTable 3. **Associations of Poor Prognostic Understanding, Prognostic Discordance, and Individual Covariates With Hospice Use **eTable 4. **Sensitivity Analyses Showing Bivariate and Multivariable Associations of Beliefs Regarding Cancer Curability and Life Expectancy Estimates With Hospitalization and Hospice Use **eTable 5. **Sensitivity Analyses Showing Bivariate and Multivariable Associations of Prognostic Discordance Regarding Curability and Survival Estimates With Hospitalization and Hospice Use

Supplement 2.
Data Sharing Statement

## References

[1] Weeks JC, Cook EF, O’Day SJ, . Relationship between cancer patients’ predictions of prognosis and their treatment preferences. JAMA. 1998;279(21):1709-1714. doi:10.1001/jama.279.21.1709 9624023

[2] Mack JW, Weeks JC, Wright AA, Block SD, Prigerson HG. End-of-life discussions, goal attainment, and distress at the end of life: predictors and outcomes of receipt of care consistent with preferences. J Clin Oncol. 2010;28(7):1203-1208. doi:10.1200/JCO.2009.25.4672 20124172 PMC2834470

[3] Enzinger AC, Zhang B, Schrag D, Prigerson HG. Outcomes of prognostic disclosure: associations with prognostic understanding, distress, and relationship with physician among patients with advanced cancer. J Clin Oncol. 2015;33(32):3809-3816. doi:10.1200/JCO.2015.61.9239 26438121 PMC4737862

[4] Mack JW, Walling A, Dy S, . Patient beliefs that chemotherapy may be curative and care received at the end of life among patients with metastatic lung and colorectal cancer. Cancer. 2015;121(11):1891-1897. doi:10.1002/cncr.29250 25677655 PMC4441582

[5] Tang ST, Liu TW, Chow JM, . Associations between accurate prognostic understanding and end-of-life care preferences and its correlates among Taiwanese terminally ill cancer patients surveyed in 2011-2012. Psychooncology. 2014;23(7):780-787. doi:10.1002/pon.3482 24470441

[6] Applebaum AJ, Kolva EA, Kulikowski JR, . Conceptualizing prognostic awareness in advanced cancer: a systematic review. J Health Psychol. 2014;19(9):1103-1119. doi:10.1177/1359105313484782 24157936 PMC4665620

[7] Gramling R, Fiscella K, Xing G, . Determinants of patient-oncologist prognostic discordance in advanced cancer. JAMA Oncol. 2016;2(11):1421-1426. doi:10.1001/jamaoncol.2016.1861 27415765 PMC5896571

[8] Loh KP, Mohile SG, Lund JL, . Beliefs about advanced cancer curability in older patients, their caregivers, and oncologists. Oncologist. 2019;24(6):e292-e302. doi:10.1634/theoncologist.2018-0890 31015317 PMC6656513

[9] Kalsi T, Babic-Illman G, Ross PJ, . The impact of comprehensive geriatric assessment interventions on tolerance to chemotherapy in older people. Br J Cancer. 2015;112(9):1435-1444. doi:10.1038/bjc.2015.120 25871332 PMC4453673

[10] Numico G, Anfossi M, Bertelli G, . The process of truth disclosure: an assessment of the results of information during the diagnostic phase in patients with cancer. Ann Oncol. 2009;20(5):941-945. doi:10.1093/annonc/mdn709 19150944

[11] Brokalaki EI, Sotiropoulos GC, Tsaras K, Brokalaki H. Awareness of diagnosis, and information-seeking behavior of hospitalized cancer patients in Greece. Support Care Cancer. 2005;13(11):938-942. doi:10.1007/s00520-005-0794-7 15800770

[12] Caruso A, Di Francesco B, Pugliese P, Cinanni V, Corlito A. Information and awareness of diagnosis and progression of cancer in adult and elderly cancer patients. Tumori. 2000;86(3):199-203. doi:10.1177/030089160008600304 10939598

[13] Henselmans I, Smets EMA, Han PKJ, de Haes HCJC, Laarhoven HWMV. How long do I have? observational study on communication about life expectancy with advanced cancer patients. Patient Educ Couns. 2017;100(10):1820-1827. doi:10.1016/j.pec.2017.05.012 28511804

[14] Loh KP, Mohile SG, Epstein RM, . Willingness to bear adversity and beliefs about the curability of advanced cancer in older adults. Cancer. 2019;125(14):2506-2513. doi:10.1002/cncr.32074 30920646 PMC6602839

[15] Thompson LL, Temel B, Fuh CX, . Perceptions of medical status and treatment goal among older adults with advanced cancer. J Geriatr Oncol. 2020;11(6):937-943. doi:10.1016/j.jgo.2019.11.005 31813839

[16] El-Jawahri A, Nelson-Lowe M, VanDusen H, . Patient-clinician discordance in perceptions of treatment risks and benefits in older patients with acute myeloid leukemia. Oncologist. 2019;24(2):247-254. doi:10.1634/theoncologist.2018-0317 30139841 PMC6369944

[17] Schnadig ID, Fromme EK, Loprinzi CL, . Patient-physician disagreement regarding performance status is associated with worse survivorship in patients with advanced cancer. Cancer. 2008;113(8):2205-2214. doi:10.1002/cncr.23856 18780322 PMC3580230

[18] Coran JJ, Koropeckyj-Cox T, Arnold CL. Are physicians and patients in agreement? exploring dyadic concordance. Health Educ Behav. 2013;40(5):603-611. doi:10.1177/1090198112473102 23345336

[19] Kerse N, Buetow S, Mainous AG III, Young G, Coster G, Arroll B. Physician-patient relationship and medication compliance: a primary care investigation. Ann Fam Med. 2004;2(5):455-461. doi:10.1370/afm.139 15506581 PMC1466710

[20] Dietrich E, Davis K, Chacko L, . Comparison of factors identified by patients and physicians associated with hospital readmission (COMPARE2). South Med J. 2019;112(4):244-250. doi:10.14423/SMJ.0000000000000959 30943545

[21] Mohile SG, Epstein RM, Hurria A, . Communication with older patients with cancer using geriatric assessment: a cluster-randomized clinical trial from the National Cancer Institute Community Oncology Research Program. JAMA Oncol. 2020;6(2):196-204. doi:10.1001/jamaoncol.2019.4728 31697365 PMC6865234

[22] Moher D, Schulz KF, Altman D; CONSORT Group (Consolidated Standards of Reporting Trials). The CONSORT statement: revised recommendations for improving the quality of reports of parallel-group randomized trials. JAMA. 2001;285(15):1987-1991. doi:10.1001/jama.285.15.1987 11308435

[23] Epstein RM, Duberstein PR, Fenton JJ, . Effect of a patient-centered communication intervention on oncologist-patient communication, quality of life, and health care utilization in advanced cancer: the VOICE randomized clinical trial. JAMA Oncol. 2017;3(1):92-100. doi:10.1001/jamaoncol.2016.4373 27612178 PMC5832439

[24] Damodaran S, Kyriakopoulos CE, Jarrard DF. Newly diagnosed metastatic prostate cancer: has the paradigm changed? Urol Clin North Am. 2017;44(4):611-621. doi:10.1016/j.ucl.2017.07.008 29107277 PMC6402766

[25] Pulte D, Castro FA, Jansen L, ; GEKID Cancer Survival Working Group. Trends in survival of chronic lymphocytic leukemia patients in Germany and the USA in the first decade of the twenty-first century. J Hematol Oncol. 2016;9:28. doi:10.1186/s13045-016-0257-2 27000264 PMC4802710

[26] Wilkowski R, Wolf M, Heinemann V. Primary advanced unresectable pancreatic cancer. Recent Results Cancer Res. 2008;177:79-93. doi:10.1007/978-3-540-71279-4_10 18084950

[27] Mohile SG, Epstein RM, Hurria A, et al. Improving communication with older patients with cancer using geriatric assessment (GA): a University of Rochester NCI Community Oncology Research Program (NCORP) cluster randomized controlled trial (CRCT). J Clin Oncol. 2018;36(18)(suppl):LBA10003. doi:10.1200/JCO.2018.36.18_suppl/LBA10003

[28] Fisher K, Seow H, Cohen J, Declercq A, Freeman S, Guthrie DM. Patient characteristics associated with prognostic awareness: a study of a Canadian palliative care population using the InterRAI palliative care instrument. J Pain Symptom Manage. 2015;49(4):716-725. doi:10.1016/j.jpainsymman.2014.08.008 25220047

[29] Johansson BB, Holmberg L, Berglund IG, Sjödén PO, Glimelius BL. Determinants of cancer patients’ utilization of hospital care within two years after diagnosis. Acta Oncol. 2004;43(6):536-544. doi:10.1080/02841860410015631 15370610

[30] Keating NL, Herrinton LJ, Zaslavsky AM, Liu L, Ayanian JZ. Variations in hospice use among cancer patients. J Natl Cancer Inst. 2006;98(15):1053-1059. doi:10.1093/jnci/djj298 16882942

[31] Vlckova K, Tuckova A, Polakova K, Loucka M. Factors associated with prognostic awareness in patients with cancer: a systematic review. Psychooncology. 2020;29(6):990-1003. doi:10.1002/pon.5385 32285580

[32] Wadhwa D, Popovic G, Pope A, Swami N, Le LW, Zimmermann C. Factors associated with early referral to palliative care in outpatients with advanced cancer. J Palliat Med. 2018;21(9):1322-1328. doi:10.1089/jpm.2017.0593 29630413

[33] Barclay JS, Kuchibhatla M, Tulsky JA, Johnson KS. Association of hospice patients’ income and care level with place of death. JAMA Intern Med. 2013;173(6):450-456. doi:10.1001/jamainternmed.2013.2773 23420383 PMC3889123

[34] Masucci L, Guerriere DN, Zagorski B, Coyte PC. Predictors of health service use over the palliative care trajectory. J Palliat Med. 2013;16(5):524-530. doi:10.1089/jpm.2012.0199 23437813

[35] Fessele KL, Hayat MJ, Atkins RL. Predictors of unplanned hospitalizations in patients with nonmetastatic lung cancer during chemotherapy. Oncol Nurs Forum. 2017;44(5):E203-E212. doi:10.1188/17.ONF.E203-E212 28820513 PMC5856246

[36] Silveira MJ, Connor SR, Goold SD, McMahon LF, Feudtner C. Community supply of hospice: does wealth play a role? J Pain Symptom Manage. 2011;42(1):76-82. doi:10.1016/j.jpainsymman.2010.09.016 21429702

[37] Davies JM, Sleeman KE, Leniz J, . Socioeconomic position and use of healthcare in the last year of life: a systematic review and meta-analysis. PLoS Med. 2019;16(4):e1002782. doi:10.1371/journal.pmed.1002782 31013279 PMC6478269

[38] Chen CH, Kuo SC, Tang ST. Current status of accurate prognostic awareness in advanced/terminally ill cancer patients: systematic review and meta-regression analysis. Palliat Med. 2017;31(5):406-418. doi:10.1177/0269216316663976 27492160

[39] Diamond EL, Corner GW, De Rosa A, Breitbart W, Applebaum AJ. Prognostic awareness and communication of prognostic information in malignant glioma: a systematic review. J Neurooncol. 2014;119(2):227-234. doi:10.1007/s11060-014-1487-1 24874468 PMC5116439

[40] Russell BJ, Ward AM. Deciding what information is necessary: do patients with advanced cancer want to know all the details? Cancer Manag Res. 2011;3:191-199. doi:10.2147/CMR.S12998 21792328 PMC3139480

[41] Duberstein PR, Chen M, Chapman BP, . Fatalism and educational disparities in beliefs about the curability of advanced cancer. Patient Educ Couns. 2018;101(1):113-118. doi:10.1016/j.pec.2017.07.007 28716485 PMC5732080

[42] Malhotra K, Fenton JJ, Duberstein PR, . Prognostic accuracy of patients, caregivers, and oncologists in advanced cancer. Cancer. 2019;125(15):2684-2692. doi:10.1002/cncr.32127 31034597

[43] Gripp S, Moeller S, Bölke E, . Survival prediction in terminally ill cancer patients by clinical estimates, laboratory tests, and self-rated anxiety and depression. J Clin Oncol. 2007;25(22):3313-3320. doi:10.1200/JCO.2006.10.5411 17664480

[44] Sun JK, Kim ES, Smith J. Positive self-perceptions of aging and lower rate of overnight hospitalization in the US population over age 50. Psychosom Med. 2017;79(1):81-90. doi:10.1097/PSY.0000000000000364 27359184 PMC5182096

[45] Okunrintemi V, Spatz ES, Di Capua P, . Patient–provider communication and health outcomes among individuals with atherosclerotic cardiovascular disease in the United States: Medical Expenditure Panel Survey 2010 to 2013. Circ Cardiovasc Qual Outcomes. 2017;10(4):e003635. doi:10.1161/CIRCOUTCOMES.117.003635 28373270

[46] Nipp RD, Greer JA, El-Jawahri A, . Coping and prognostic awareness in patients with advanced cancer. J Clin Oncol. 2017;35(22):2551-2557. doi:10.1200/JCO.2016.71.3404 28574777 PMC5536163

[47] Loh KP, Mohamed MR, Kadambi S, . Caregiver–oncologist prognostic concordance, caregiver mastery, and caregiver psychological health and quality of life. Oncologist. 2021;26(4):310-317. doi:10.1002/onco.13699 33523583 PMC8018313

[48] Temel JS, Greer JA, Admane S, . Longitudinal perceptions of prognosis and goals of therapy in patients with metastatic non–small-cell lung cancer: results of a randomized study of early palliative care. J Clin Oncol. 2011;29(17):2319-2326. doi:10.1200/JCO.2010.32.4459 21555700

