# Supplementary material for: Association of Prognostic Understanding With Health Care Use Among Older Adults With Advanced Cancer: A Secondary Analysis of a Cluster Randomized Clinical Trial
Source: JAMA Netw Open. 2022 Feb 18;5(2):e220018. doi: 10.1001/jamanetworkopen.2022.0018 (PMC8857680; doi:10.1001/jamanetworkopen.2022.0018)
Supplement: Supplement 1. — eTable 1. National Cancer Institute Community Oncology Research Program (NCORP) Community Affiliate Partners eTable 2. Associations of Poor Prognostic Understanding, Prognostic Discordance, and Individual Covariates With Hospitalization at 6 Months eTable 3. Associations of Poor Prognostic Understanding, Prognostic Discordance, and Individual Covariates With Hospice Use eTable 4. Sensitivity Analyses Showing Bivariate and Multivariable Associations of Beliefs Regarding Cancer Curability and Life Expectancy Estimates With Hospitalization and Hospice Use eTable 5. Sensitivity Analyses Showing Bivariate and Multivariable Associations of Prognostic Discordance Regarding Curability and Survival Estimates With Hospitalization and Hospice Use [file jamanetwopen-e220018-s001.pdf]

Supplementary Online Content

Loh KP, Seplaki CL, Sanapala C, et al. Association of prognostic understanding with health care use among older adults with advanced cancer: a secondary analysis of a cluster randomized clinical trial. *JAMA Netw Open.* 2022;5(2):e220018. doi:10.1001/jamanetworkopen.2022.0018

eTable 1. National Cancer Institute Community Oncology Research Program (NCORP) Community Affiliate Partners

eTable 2. Associations of Poor Prognostic Understanding, Prognostic Discordance, and Individual Covariates With Hospitalization at 6 Months

eTable 3. Associations of Poor Prognostic Understanding, Prognostic Discordance, and Individual Covariates With Hospice Use

eTable 4. Sensitivity Analyses Showing Bivariate and Multivariable Associations of Beliefs Regarding Cancer Curability and Life Expectancy Estimates With Hospitalization and Hospice Use

eTable 5. Sensitivity Analyses Showing Bivariate and Multivariable Associations of Prognostic Discordance Regarding Curability and Survival Estimates With Hospitalization and Hospice Use

This supplementary material has been provided by the authors to give readers additional information about their work.

eTable 1. National Cancer Institute Community Oncology Research Program (NCORP) Community Affiliate Partners

**NCORP Community Affiliate**
Aurora NCORP (Illinois, Wisconsin)
Columbus NCORP (Columbus, Ohio)
Delaware/Christiana Care (Newark, Delaware)
Geisinger Cancer Institute Oncology Research Program (Danville, Pennsylvania)
NCORP of the Carolinas (Greenville, South Carolina)
Hawaii Minority Underserved NCORP (Honolulu, Hawaii)
Heartland Cancer Research NCORP (Decatur, Illinois)
Metro-Minnesota NCORP (Saint Louis Park, Minnesota)
Michigan Cancer Research Consortium NCORP (Ann Arbor, Michigan)
Nevada Cancer Research Foundation NCORP (Las Vegas, Nevada)
Northwell Health NCORP (Lake Success, New York)
Pacific Cancer Research Consortium NCORP (Seattle, Washington)
Southeast Clinical Oncology Consortium NCORP (Winston- Salem, North Carolina)
Wisconsin NCORP (Marshfield, Wisconsin)
Wichita NCORP (Wichita, Kansas)

© 2022 Loh KP et al. *JAMA Network Open.*

eTable 2. Associations of Poor Prognostic Understanding, Prognostic Discordance, and Individual Covariates With Hospitalization at 6 Months

		No hospitalization (N=411)	Hospitalization (N=130)	*P* value
Age in years, mean (SD)		76.6 (5.3)	76.5 (5.1)	0.95
Gender[a]	Male	204 (49.8)	72 (55.4)	0.26
	Female	206 (50.2)	58 (44.6)	
Race[a]	Black	34 (8.3)	6 (4.6)	0.10
	White	368 (89.8)	118 (90.8)	
	Other[g]	8 (2.0)	6 (4.6)	
Education[a]	<High school	51 (12.4)	15 (11.5)	0.70
	High school graduate	144 (35.1)	51 (39.2)	
	Some college or above	215 (52.4)	64 (49.2)	
Marital status[a]	Married	267 (65.1)	81 (62.3)	0.56
	Other	143 (34.9)	49 (37.7)	
Income[b]	≤$50,000	194 (47.6)	71 (54.6)	0.34
	>$50,000	127 (31.1)	37 (28.5)	
	Decline to answer	87 (21.3)	22 (16.9)	
Cancer type[a]	Breast	57 (13.9)	13 (10.1)	0.41
	Gastrointestinal	91 (22.1)	38 (27.1)	
	Genitourinary	55 (13.4)	17 (13.2)	
	Lung	100 (24.3)	37 (28.7)	
	Other	108 (26.3)	27 (20.9)	
Impaired geriatric assessment domain	Physical performance	386 (93.9)	121 (93.1)	0.73
	Functional status	235 (57.2)	84 (64.6)	0.13
	Comorbidity	251 (61.1)	94 (72.3)	0.02
	Nutrition	246 (59.9)	80 (61.5)	0.73
	Cognition	140 (35.2)	40 (28.0)	0.009
	Medical social support	108 (26.5)	47 (36.2)	0.03
	Psychological status	97 (23.6)	39 (30.0)	0.14
	Polypharmacy	344 (83.7)	109 (83.9)	0.97
Poor prognostic understanding regarding curability[c]		161 (63.1)	45 (52.9)	0.17
Poor prognostic understanding		163 (43.6)	41 (34.4)	0.07

© 2022 Loh KP et al. *JAMA Network Open.*

regarding life expectancy estimates[d]				
Prognostic discordance regarding curability[e]		144 (61.5)	47 (56.0)	0.37
Prognostic discordance regarding life expectancy estimates[f]		261 (70.5)	95 (77.9)	0.12

[a]Missing data in 1
[b]Missing data in 3
[c]Missing data in 193
[d]Missing data in 45
[e]Missing data in 205
[f]Missing data in 49
[g]7 patients were Asian, 4 patients were American Indian/Alaskan Native, 1 patient was Native Hawaiian or Other Pacific Islander, 2 patients had mixed races

© 2022 Loh KP et al. *JAMA Network Open.*

eTable 3. Associations of Poor Prognostic Understanding, Prognostic Discordance, and Individual Covariates With Hospice Use

		Not enrolled on hospice (N=459)	Enrolled on hospice (N=82)	*P* value
Age in years, mean (SD)		76.6 (5.2)	76.6 (5.6)	0.02
Gender[a]	Male	232 (50.7)	44 (53.7)	0.62
	Female	226 (49.3)	38 (46.3)	
Race[a]	Black	38 (8.3)	2 (2.4)	0.17
	White	408 (89.1)	78 (95.1)	
	Other	12 (2.6)	2 (2.4)	
Education[a]	<High school	51 (11.1)	15 (18.3)	0.09
	High school graduate	172 (37.6)	23 (28.1)	
	Some college or above	235 (51.3)	44 (54.7)	
Marital status[a]	Married	300 (65.5)	48 (58.5)	0.22
	Other	159 (34.5)	34 (41.5)	
Income[b]	≤$50,000	225 (49.3)	40 (48.8)	<0.01
	>$50,000	148 (32.5)	16 (19.5)	
	Decline to answer	83 (18.2)	26 (31.7)	
Cancer type[a]	Breast	67 (14.6)	3 (3.7)	<0.01
	Gastrointestinal	93 (20.3)	33 (40.7)	
	Genitourinary	70 (15.3)	2 (2.5)	
	Lung	111 (24.2)	26 (32.1)	
	Other[g]	118 (25.7)	17 (21.0)	
Impaired geriatric assessment domain	Physical performance	430 (93.7)	77 (93.9)	0.94
	Functional status	252 (54.9)	67 (81.7)	<0.01
	Comorbidity	286 (62.3)	59 (72.0)	0.09
	Nutrition	259 (56.4)	67 (81.7)	<0.01
	Cognition	153 (33.3)	27 (32.9)	0.94
	Instrumental social support	122 (26.6)	34 (41.5)	<0.01
	Psychological status	105 (22.9)	31 (37.8)	<0.01
	Polypharmacy	388 (84.5)	65 (79.3)	0.23
Poor prognostic understanding regarding curability[c]	Yes	178 (60.8)	28 (50.9)	0.17
Poor prognostic understanding regarding	Yes	192 (45.6)	13 (17.3)	<0.01

© 2022 Loh KP et al. *JAMA Network Open.*

life expectancy estimates[d]				
Prognostic discordance regarding curability[e]	Yes	172 (61.2)	30 (54.6)	0.36
Prognostic discordance regarding life expectancy estimates[f]	Yes	299 (71.7)	57 (76.0)	0.44

[a]Missing data in 1
[b]Missing data in 2
[c]Missing data in 193
[d]Missing data in 45
[e]Missing data in 205
[f]Missing data in 49
[g]7 patients were Asian, 4 patients were American Indian/Alaskan Native, 1 patient was Native Hawaiian or Other Pacific Islander, 2 patients had mixed races

© 2022 Loh KP et al. *JAMA Network Open.*

eTable 4. Sensitivity Analyses Showing Bivariate and Multivariable Associations of Beliefs Regarding Cancer Curability and Life Expectancy Estimates With Hospitalization and Hospice Use

		**Bivariate Analyses[a]**	**Multivariable Analyses[b]**
		**Odds Ratio (95% Confidence Interval)**	**Adjusted Odds Ratio (95% Confidence Interval)**
	Hospitalization		
Beliefs about curability	0%	Ref	Ref
	<50%	0.81 (0.55-1.18)	0.66 (0.36-1.20)
	50%	0.86 (0.41-1.77)	0.84 (0.45-1.59)
	>50%	0.47 (0.22-1.01)	0.62 (0.21-1.22)
	100%	1.00 (0.37-2.71)	1.01 (0.41-2.49)
Life expectancy estimates	0-6 months	Ref	Ref
	7-12 months	0.78 (0.18-3.29)	0.69 (0.15-3.25)
	1-2 years	0.63 (0.16-2.54)	0.73 (0.17-3.24)
	2-5 years	0.85 (0.19-3.79)	1.18 (0.24-5.86)
	>5 years	0.51 (0.14-1.82)	0.72 (0.20-2.59)
	Hospice Use		
Beliefs about curability	0%	Ref	Ref
	<50%	1.05 (0.55-1.98)	1.18 (0.62-2.23)
	50%	0.54 (0.24-1.19)	0.43 (0.17-1.09)
	>50%	0.43 (0.21-0.89)	0.69 (0.30-1.60)
	100%	0.71 (0.11-4.70)	0.54 (0.06-4.72)
Life expectancy estimates	0-6 months	Ref	Ref
	7-12 months	1.83 (0.36-9.28)	2.00 (0.45-8.81)
	1-2 years	0.66 (0.15-2.88)	0.70 (0.18-2.64)
	2-5 years	0.30 (0.07-1.33)	0.59 (0.16-2.20)
	>5 years	0.14 (0.04-0.46)	0.21 (0.07-0.66)

[a]Accounting for clustering at the practice level
[b]Adjusted for demographics, cancer type, study arm, and age-related conditions and accounting for clustering at the practice level

© 2022 Loh KP et al. *JAMA Network Open.*

eTable 5. Sensitivity Analyses Showing Bivariate and Multivariable Associations of Prognostic Discordance Regarding Curability and Survival Estimates With Hospitalization and Hospice Use

	**Bivariate Analyses[a]**	**Multivariable Analyses[b]**
	**Odds Ratio (95% Confidence Interval)**	**Adjusted Odds Ratio (95% Confidence Interval)**
Hospitalization		
Prognostic discordance regarding curability[c]	0.74 (0.45-1.24)	0.71 (0.44-1.14)
Prognostic discordance regarding life expectancy estimates[d]	1.67 (1.08-2.59)	1.73 (1.03-2.93)
Hospice Use		
Prognostic discordance regarding curability[c]	0.98 (0.63-1.53)	0.89 (0.55-1.42)
Prognostic discordance regarding life expectancy estimates[d]	1.88 (1.24-2.85)	1.46 (0.87-2.43)

[a]Accounting for clustering at the practice level

[b]Adjusted for demographics, cancer type, study arm, and age-related conditions and accounting for clustering at the practice level

[c]Beliefs about curability were collapsed in the following ways for both patients and oncologists: 0% vs. >0 to 100%. Prognostic discordance regarding curability was defined if there was any patient-oncologist difference in the response options

[d]Life expectancy estimates were collapsed in the following ways for both patients and oncologists: 0-12 months vs. 1-2 years vs. 2 to >5 years. Prognostic discordance regarding life expectancy estimates was defined if there was any patient-oncologist difference in the response options

© 2022 Loh KP et al. *JAMA Network Open.*
