# Supplementary material for: Association of Prognostic Understanding With Health Care Use Among Older Adults With Advanced Cancer: A Secondary Analysis of a Cluster Randomized Clinical Trial
Source: JAMA Netw Open. 2022 Feb 18;5(2):e220018. doi: 10.1001/jamanetworkopen.2022.0018 (PMC8857680; doi:10.1001/jamanetworkopen.2022.0018)
Supplement: Supplement 2. — Data Sharing Statement [file jamanetwopen-e220018-s002.pdf]

Data Sharing Statement

Loh. Association of Prognostic Understanding With Health Care Use Among Older Adults With Advanced Cancer. *JAMA Netw Open*. Published February 18, 2022.
doi:10.1001/jamanetworkopen.2022.0018

Data
Data available: Yes
Data types: Other (please specify)
Additional Information: Available on request
How to access data: kahpoh_loh@urmc.rochester.edu
When available: With publication

Supporting Documents
Document types: None

Additional Information
Who can access the data: The trial protocol has been previously published in the parent study
Types of analyses: The trial protocol has been previously published in the parent study
Mechanisms of data availability: Already published
